# Single-cell sequencing reveals cellular heterogeneity and molecular mechanisms in tendon and enthesis injury repair

**DOI:** 10.3389/fphys.2025.1685955

**Published:** 2025-10-29

**Authors:** Tingming Pan, Zhong Dong, Hongjie Zhang, Fengmin Yang, Yating Chen

**Affiliations:** Department of Orthopedics, Fujian Provincial Second People’s Hospital, The Second Affiliated Hospital of Fujian University of Traditional Chinese Medicine, Fuzhou, China

**Keywords:** tendon healing, enthesis healing, single-cell sequencing, immune regulation, cellular interactions

## Abstract

**Background:**

Tendon and enthesis injuries represent a significant global health challenge, severely impairing patient mobility and self-care abilities while imposing substantial medical burdens.

**Main Body:**

Poor clinical outcomes in tendon healing stem from the complex enthesis, which involves diverse cell types and signaling pathways. Recent advances in single-cell sequencing technologies have revealed detailed cellular diversity and function in tendon and tendon-bone healing. Using multimodal integration, researchers have identified precise subpopulations of tendon and enthesis cells. They have also clarified cell-to-cell crosstalk and mapped differentiation paths during healing.

**Conclusion:**

These new findings, guided by emerging methodological advancements. They offer innovative perspectives for developing targeted clinical interventions for tendon and enthesis injury.

## 1 Background

Tendons are dense fibrous tissues connecting muscles to bones and play a critical role in the musculoskeletal system by resisting tensile forces and bearing mechanical loads. Each tendon attaches at one end to skeletal muscle and at the other to bone, creating a transitional zone between hard and soft tissues. This zone, known as the enthesis, features a gradual transition in tissue organization (Zhou H. et al., 2025). Trauma or age-related degeneration of tendons and the enthesis can cause rupture or damage, significantly impairing mobility and leading to disability. Tendon and enthesis injuries are common in orthopedic practice, accounting for approximately one-third of cases and resulting in significant economic costs. (Wang M. et al., 2025). In the United States, over 300,000 tendon injury cases are reported annually (Jiang F. et al., 2024), and in New Zealand, nearly 200,000 incidents are reported annually, with direct economic burdens exceeding 300 million USD (Clark et al., 2020). Despite the high incidence, successful repair remains challenging, with procedures such as rotator cuff repairs showing recurrence rates up to 94% within 2 years (Qian et al., 2024). Anterior cruciate ligament reconstructions also have frequent primary surgical failures (Rodriguez Merchan, 2025). Among young patients, more than 10% experience long-term functional decline or reconstruction failure (Migliorini et al., 2024), rising to over one-third in high-intensity athletes (Winkler et al., 2025).

Global bibliometric analyses confirm an accelerating interest in tendon stem/progenitor cells (TSPCs) biology as a cornerstone of musculoskeletal regeneration (Zhang S. et al., 2023). A growing body of research has demonstrated that the healing process of tendons and the enthesis is influenced by multiple factors, with cellular heterogeneity and subsequent intercellular interactions play a significant role in determining healing outcomes (Best and Loiselle, 2019; Ackerman et al., 2021; Korcari et al., 2022). Notably, phenotypic and functional alterations in immune cells following injury, as well as differentiation pathways of stem/progenitor cells, play crucial roles. However, conventional histological methods, which rely on population-averaged analyses (e.g., PCR, RNA-seq),face limitations in capturing the heterogeneity and dynamic changes of critical cellular subpopulations within the tendon injury microenvironment, potentially obscuring the functional contributions of key cell types (Tong et al., 2023). Such technical constraints substantially hinder comprehensive understanding and clinical translation of tendon and enthesis regeneration. Recent studies have revealed that tendon-derived stem cells and tenocytes comprise functionally diverse subpopulations, highlighting that investigations lacking single-cell resolution cannot provide biologically authentic evidence for clinical therapeutic development. Consequently, systematic characterization of cellular composition and functional dynamics within the tendon-bone healing microenvironment has emerged as a critical research priority.

**FIGURE 1:**
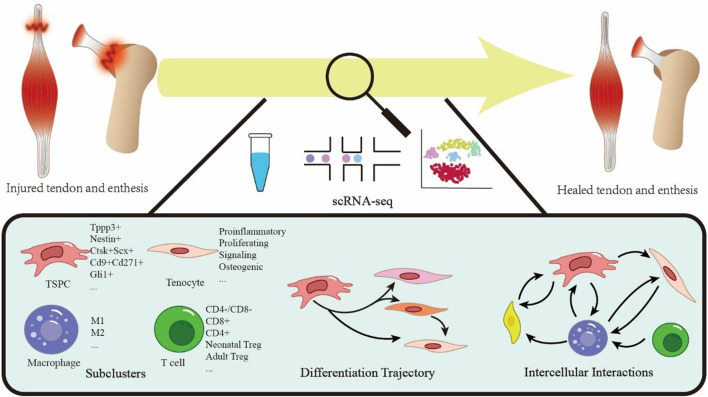
Schematic overview of single-cell sequencing technology applied to tendon and enthesis healing.

Single-cell RNA sequencing (scRNA-seq) offers transformative potential in addressing these challenges. The workflow includes tissue dissociation, cell capture andlysis, RNA extraction, reverse transcription to cDNA, amplification, library preparation, and reconstruction of single-cell transcriptional profiles (Saleh et al., 2025). Figure 1 visualizes this pipeline. This approach enables the resolution of cellular heterogeneity-critical for tendon healing studies-and overcomes technical challenges in dense, collagen-rich tendon samples, thereby helping to decipher repair mechanisms. The technology has evolved from basic scRNA-seq to spatial transcriptomics, providing precise resolution of cellular composition and interactions at the single-cell level. Compared to conventional methods, scRNA-seq has two major advantages: revealing cellular heterogeneity and subclusters, as well as mapping cellular states, transitional trajectories, and differentiation pathways in physiological orpathological processes (Wang T. et al., 2023). In orthopedics research, scRNA-seq has provided significant insights. Li et al. showed macrophages synergize with glucocorticoids to promote osteogenesis, while excess glucocorticoids disrupt local fatty acid transport in macrophages and impair bone turnover (Li et al., 2024). In osteoarthritis research, Liu et al. found Angptl7+ chondrocytes drive H-type vessel formation through Fgf2-Fgfr2 signaling in endothelial cells. Dysfunctional mineralization of Sparc + osteoblasts contributes to subchondral bone remodeling (Liu Y. et al., 2025), revealing potential therapeutic targets. The core strength of scRNA-seq lies in its ability to unravel cellular heterogeneity and microenvironment dynamics, providingmolecular insights that advance disease diagnosis, optimize therapeutic strategies, and aid in drug development (Song W. et al., 2023; Li et al., 2025a).

In tendon research, scRNA-seq technology has advanced healing studies at the enthesis, revealing key discoveries that address critical knowledge gaps. Compared to traditional methods, scRNA-seq has facilitated the mapping of TSPCs differentiation, characterized stromal cell subtypes, and clarified interaction between immune and tendon-lineage cells (Liu W. et al., 2025). These findings provide essential insights into molecular underlying repair and support the development of novel therapeutic strategies.

Leveraging single-cell data holds the potential to revolutionize the clinical management of tendon and enthesis healing. Existing reviews focus on cell types or static molecular mechanisms, often missing a comprehensive analysis of cellular dynamics during repair. They also do not systematically assess spatiotemporal (both spatial and temporal) healing. Therefore, this review addresses three key aspects: 1) Breakthroughs and platform choices in scRNA-seq for tendon healing research; 2) Tendon injury repair mechanisms elucidated through scRNA-seq analysis; 3) Tendon-bone healing explored using scRNA-seq technology. This review synthesizes current experimental evidence and provide insights for clinical translation strategies.

## 2 Comprehensive workflow analysis of single-cell technologies in tendon-bone healing

### 2.1 Current challenges and pitfalls in scRNA-seq for tendon research

ScRNA-seq requires rigorous sample preparation and methodological optimization. Tendon tissues possess a dense collagenous structure. Type I collagen comprises approximately 86% of the content, and the extracellular matrix is rigid, which prevents conventional enzymatic digestion protocols from efficiently releasing functional cells. The dissociation process often generates filamentous collagen residues. These residues compromise droplet capture efficiency (Autengruber et al., 2012; Reichard and Asosingh, 2019). Simultaneously, mechanical shear forces may induce aberrant expression of stress-response genes. This introduces transcriptomic bias (Su et al., 2024). Furthermore, the enthesis is a heterogeneous transition zone with tendon, fibrocartilage, and bone tissues. Achieving dissociation homogeneity is challenging due to physicochemical differences among the cell populations (tenocytes, chondrocytes, osteoblasts). This may lead to loss or enrichment bias of specific subpopulations such as CD26^+^ TSPCs (Chen et al., 2025). Tendon tissues or enthesis are inherently low in cell numbers. Sufficient cell numbers (>10^4) are needed to adequately represent rare subpopulations. However, clinical tendon biopsy specimens typically weigh only 50–100 mg. Fibrotic or calcified lesions in injured tissues further reduces viable cell yields (Zhang T. et al., 2023). Moreover, healing in tendons and entheses is dynamically regulated by multicellular crosstalk. Analyses limited to a single time point or focusing only on one cell type do not fully capture or resolve the healing process. This imposes major demands on scRNA-seq analytical frameworks and multimodal data integration (Jiang Z. et al., 2024). Technical hurdles in workflows and data interpretation are major bottlenecks in tendon and enthesis research. Recent advances are enabling the more accessible and precise use of scRNA-seq in these studies. Nevertheless, limitations persist in applying scRNA-seq to tendon biology. First, enzymatic dissociation methods underrepresent low-abundance cell types (e.g., Tppp3+ progenitors, tissue-resident immune subsets) and introduce capture bias. This can obscure rare functional population. Second, scRNA-seq cannot resolve the dynamics of extracellular matrix (ECM) protein. Transcript levels do not reflect post-translational modifications of collagen isoforms, which are fundamental for tendon mechanics. Third, current platforms cannot simulate *in vivo* mechanical stimuli that regulate tenocyte phenotypes. This limits insights into load-induced pathologies, such as tendinopathy or ectopic ossification.

To overcome these constraints, three synergistic strategies should be prioritized. First is multi-omics integration. Combining scRNA-seq with proteomics, such as CITE-seq, can validate the expression of ECM proteins. This approach helps resolve discordance between mRNA and protein-level regulation (Fetahu I. S. et al., 2023). Second, spatial transcriptomics enables spatial mapping of cellular niches within tendon-bone interfaces. Technologies like Visium reveal zonal heterogeneity in enthesis progenitors and capture niche-specific ECM cues (Zhang T. et al., 2023). Third, organoid-based validation is essential. Species divergence in mechanosensitive genes, such as ACAN and COL1A1, necessitates the use of human tendon organoids to test therapeutics. These models better capture load-induced changes than rodent systems (Su W. et al., 2024; He Z. et al., 2025).

### 2.2 Specific requirements for sample preparation

Sample preparation is critical in single-cell sequencing. Tendon tissue is relatively homogeneous and easy to dissociate, but enthesis presents challenges due to its complex structure, varied density, and diverse cell types. Isolating distinct cell types without functional loss remains a significant challenge. Thin cellular layers at the tendon-bone junction have also made precise sampling historically challenging (Zhang T. et al., 2023).

Traditional methods for mechanically breaking up tissues can damage cell membranes, leading to significant decreases in cell energy production and alterations in the function of small cellular components called mitochondria, which can harm or kill the cells (Su et al., 2024). Using chemicals to dissolve tissue helps get more cells, but can change the types and roles of immune cells (Autengruber et al., 2012; Reichard and Asosingh, 2019). Since Given immune cells play a crucial role in how cells communicate in injured tendons, it is essential not to harm them excessively. Therefore, improving tissue processing methods-including selecting the appropriate chemicals, limiting the duration of tissue treatment, and promptly separating cells afterward-is crucial for obtaining high-quality samples (He J. et al., 2023; Xu Z. et al., 2025). The best current methods for studying tendons utilize short, gentle chemical treatment with soft shaking, followed by cell sorting that identifies rare cell types, resulting in good outcomes. (Chen et al., 2025). Enzymatic digestion is commonly used to isolate individual muscle or tendon cells, typically involving 1-h incubation with a cocktail of collagenase II, collagenase D, and dispase II (Shahini et al., 2018). Zhang et al. also noted that careful cutting should be performed first when aiming to obtain cells from specific regions where the tendon attaches to the bone (Zhang T. et al., 2023).

### 2.3 Advantages of multimodal integration

#### 2.3.1 CITE-seq (cellular indexing of transcriptomes and epitopes by sequencing) for concurrent transcriptomic and surface protein profiling of immune cells

CITE-seq is an advanced single-cell multi-omics technology that enables simultaneous profiling of gene expression and surface protein phenotypes by integrating transcriptome sequencing with antibody-oligonucleotide conjugate labeling (Lukyanov et al., 2025). The method utilizes DNA-barcoded antibodies against predefined surface antigens, transforming protein signals into DNA for sequencing during single-cell library preparation. This simultaneous capture of RNA (genetic activity) and targeted protein information at the single-cell level (Inamo et al., 2025) enables multimodal, antibody-directed analysis, which significantly enhances cell type identification. This capability is particularly valuable in immune microenvironment studies, which aim to reveal functional cell subsets that are indistinguishable by RNA sequencing alone (Wang et al., 2024). For example, CITE-seq highlights the diverse states of macrophages or different stages of T cell activation (Nettersheim et al., 2022). The main advantages of CITE-seq include: 1) integrating multiple biological data types to construct comprehensive, multidimensional cellular maps; 2) confirming cell subset identity with both gene expression and surface protein markers, thus reducing annotation errors; and 3) sensitively detecting rare cell populations, such as tissue-resident stem cells or abnormal clusters in diseased tissues. Importantly, CITE-seq analyzes only pre-selected surface markers—not all proteins—enabling sensitive immune cell profiling without implying broad discovery proteomics.

#### 2.3.2 Application of pseudotime analysis in reconstructing tendon stem cell differentiation trajectories

Pseudotime analysis is a computational method using single-cell transcriptomic data. It maps cellular similarities in gene expression onto a pseudo-temporal continuum by employing dimensionality reduction and trajectory inference to model cell differentiation or development (Canalis et al., 2025). Specifically, the method assumes that cells at various stages of differentiation coexist in a single sample. By comparing gene expression patterns, it creates trajectories from initial to terminal states, pinpointing key transition points at which major shifts in cell identity and fate decisions occurs (Gagler et al., 2025). As a tool for reconstructing differentiation trajectories, pseudotime analysis excels in tendon stem cell research; with single-cell transcriptomic data, research can infer distinct states and identify the timing of key transitions in stem cell differentiation (Shen et al., 2025). This approach not only enhances understanding of tendon stem cell transformation after injury but also reveals essential transcription factors and signaling pathways in differentiation. Its application provides a theoretical basis for developing therapeutic strategies, especially for tendon healing and regeneration (Zhang T. et al., 2023). Furthermore, combining pseudotime analysis with other single-cell methods yields a more comprehensive understanding of tendon stem cells in tissue repair.

Pseudotime analysis demonstrates an unique applicability in tendon research for two primary reasons: First, tendon stem cells exhibit well-defined heterogeneous differentiation pathways. For example, in zebrafish intermuscular bone formation studies, pseudotime analysis revealed that TSPCs bifurcate into osteogenic or mature tenocyte lineages, with key regulatory genes like Runx2b and Entpd5a identified—a finding highly relevant to post-injury fibrosis or heterotopic ossification mechanisms (Nie et al., 2022). Second, tendon repair involves complex cellular state transitions. Intermediate cell subsets, such as pro-inflammatory tenocytes in the tendon microenvironment, exhibit continuous gradient-like gene expression patterns, captured by Huang et al. using pseudotime analysis (Huang Z. et al., 2025). These characteristics establish pseudotime analysis as a robust tool for unraveling molecular mechanisms of tendon regeneration and pathological remodeling, providing theoretical foundations for targeted intervention strategies.

### 2.4 Platform selection guidelines

In orthopedic tissue-specific research, scRNA-seq technologies are critical tools for understanding tendon and tendon-bone healing mechanisms. The widely used 10× Genomics Chromium system and BD Rhapsody system, which use different cell isolation and barcoding approaches, have complementary strengths in tendon and bone interfaces. Table 1 compares these two sequencing platforms for enthesis research.

**TABLE 1:** Comparative analysis of single-cell sequencing platforms for enthesis research.

Feature	10× genomics chromium	BD rhapsody
Core Technology	Closed microfluidic droplet system (Ramarapu R. et al., 2024)	Semi-open microwell plate (CytoSeq™) (Scheiber A. et al., 2024)
Throughput	High (10^4^–10^5^ cells/run) (Ramarapu R. et al., 2024)	Moderate (≤10^4^ cells/run) (Colino-Sanguino et al., 2024)
Cell Capture Mechanism	Probability-based barcoding (Lin P. et al., 2024)	Physical microwell sedimentation (Colino-Sanguino et al., 2024)
Ionic Sensitivity	Reverse transcriptase inhibition in ion-rich entheses (Zhang T. et al., 2023)	Washable design removes EDTA/DNase I residues (Colino-Sanguino et al., 2024)
Rare Cell Recovery	Limited for CD26^+^ progenitors (Chen S. et al., 2025)	Enhanced capture of Tppp3+ TSPCs (Nielsen M. R. et al., 2024)
Ideal Application	Temporal macrophage polarization (Huang Z. et al., 2025) Stem cell trajectories (Nie C. H. et al., 2022)	Pathogenic tenocyte subsets (ADAM12hi clusters) (Mimpen J. Y. et al., 2025) Low-input biopsies (50–100 mg) (Zhang T. et al., 2023)

For tendon injury models, platform selection should prioritize biological context and sample constraints. The 10x Chromium system offers superior throughput, enabling high-resolution temporal mapping of immune dynamics (Huang Z. et al., 2025) and stem cell trajectories during healing (Ramarapu R. et al., 2024). However, it is susceptible to ionic interference in mineralized entheses (Zhang T. et al., 2023), necessitating stringent sample preprocessing. In contrast, BD Rhapsody’s washable microwell architecture enhances tolerance to enzymatic residues and ECM aggregates (Colino-Sanguino et al., 2024),making it optimal for fibrosis-dominated tendons (Nielsen M. R. et al., 2024) and for detecting rare progenitor cells, such as CD26^+^ TSPCs (Chen S. et al., 2025). When processing scarce clinical specimens (≤100 mg) (Zhang T. et al., 2023), BD’s robust impurity removal offsets moderate throughput, while 10x is preferable for comprehensive atlasing of injury phases requiring extensive cell recovery.

## 3 Single-cell sequencing-based research on tendon healing

The traditional tendon healing response comprises four key phases: 1) The immune cell response and inflammatory phase begin with a hematoma after tendon rupture. Neutrophils, monocytes, and macrophages enter the fibrin clot within hours to days. These cells removes phagocytose necrotic cells and damaged ECM, releasing pro-inflammatory cytokines and chemokines; 2) In the next 2 weeks, progenitor cells migrate to the injury site. 3) Then, TSPCs differentiate into functional cells and synthesize a provisional matrix dominated by type III collagen. Over 2 weeks to months, type I collagen gradually replaces it during remodeling; 4) The ECM remodeling phase uses mechanical stress for tissue reorganization (Kent Iii et al., 2024; Rieber et al., 2025). Alternatively, tendon healing may be categorized into three phases—inflammatory, proliferative, and remodeling—by merging matrix remodeling with mechanical maturation. This scheme highlights biological continuity and aligns with findings from single-cell sequencing (Huang Z. et al., 2025).

Throughout the healing process, tendon tissues undergo complex molecular and cellular cascades involving dynamic regulation of ECM components, cytokines, growth factors, and immune cells, which collectively shape the local healing microenvironment (Shahri et al., 2025). Notably, tendon repair outcomes are highly dependent on this microenvironmental regulation, where interactions among signaling molecules, inflammatory mediators, immune cells, and endothelial cells may lead to divergent clinical endpoints (Lin C. Y. et al., 2025; Wolint et al., 2025). Current clinical strategies predominantly rely on fibroblast-mediated scar repair mechanisms. However, outcomes remain unpredictable: heterotopic ossification represents a severe adverse event, and even in cases of anatomically continuous scar repair, reduced collagen fiber diameter, disorganized fiber alignment, and incomplete restoration of mechanical strength are common, predisposing the tissue to re-rupture (Chong et al., 2025; Rodenhouse et al., 2025; Shahri et al., 2025).

Conventional research methods often fall short in systematically addressing tendon healing mechanisms. This limits our understanding of how these mechanisms work and impedes optimal repair strategies. In contrast, recent advances in single-cell technologies allow researchers to investigate the role of individual cells in tendon injury repair.

### 3.1 Cell subtypes and their functions in normal or injured tendons revealed by single-cell sequencing

#### 3.1.1 Tendon lineage cells

##### 3.1.1.1 Stem and progenitor cells

TSPCs are a foundational cell population within tendon tissue, known for self-renewal and multipotent differentiation. Present in both human and animal tendons-such as patellar tendons, Achilles tendons, rotator cuffs in mice, rats, rabbits, and pigs) (Bi et al., 2007; He et al., 2025)- these cells are variably described as “tendon stem cells” or “tendon progenitor cells” due to their dual stem cell traits and heterogeneous functions. Their endogenous migration to injury sites and microenvironment-driven differentiation into tenocytes are key mechanisms of tendon regeneration (Ahn, 2024). Impaired TSPC activity disrupts ECM remodeling, leading to collagen fiber disorganization, reduced mechanical strength, and chronic tendinopathy (Chang et al., 2019).

TSPC identification faces significant technical challenges, primarily due to the morphological similarity between tendon stem/progenitor cells (TSPCs) and mature tenocytes, as well as their overlapping isolation protocols (Walia and Huang, 2019). The current consensus defines TSPCs by the expression of mesenchymal stem cell (MSC) markers (such as Sca-1, CD44, CD90, CD105, and CD146) and by the lack of hematopoietic/endothelial markers (CD31, CD34, CD18, CD117, and CD45) (Sakai and Kumagai, 2025). Unlike other MSCs, TSPCs uniquely express tendon-specific genes such as Scleraxis (Scx), Tenomodulin (Tnmd), and Tenascin-C (TNC) (Ahn, 2024). Single-cell transcriptomics has further refined their molecular definition. Fu et al. used markers such as ACTA2, THY1, and MCAM to identify TSPC populations (Fu et al., 2023). Lin et al. defined tendon progenitor subsets by CD44, Thy1, and Ly6a, with Itm2a marking Tenoblasts, thetransitional cells between progenitors and mature tenocytes (Lin J. et al., 2022).

These functionally diverse TSPC subpopulations (Table 2) exhibit distinct regenerative capacities. Recent studies have highlighted significant heterogeneity within TSPC populations, as demonstrated by region-dependent functional differences in cells isolated from different anatomical origins (e.g., patellar vs. Achilles tendons) (Mienaltowski et al., 2014). Furthermore, Brown and Huang demonstrated spatiotemporal specificity in TSPC responses to growth factors, showing that subsets from different developmental stages or tissues exhibit varying sensitivity to TGF-β, BMP-2, and other signals (Brown et al., 2014; Huang et al., 2021). This functional diversity, corroborated by recent tissue engineering studies (He W. et al., 2024), underscores the necessity for maker-driven purification in therapeutic applications. Collectively, these findings suggest that TSPCs comprise multiple subpopulations with distinct proliferative and differentiation potentials.

**TABLE 2:** Tendon stem/progenitor cells (TSPCs) and tenocyte subpopulations.

Cluster name	Unique markers	Function	References
Tppp3+ TSPC	Tppp3	Tendon healing and ectopic ossification	Harvey et al. (2019)
Tppp3+Pdgfra + TSPC	Tppp3, Pdgfra (no SCX)	Tendon healing and differentiation into tenocytes	Yea et al. (2023)
Nestin + TSPC	Nestin	Self-renewal and tenogenic potential; suppression of non-tenogenic differentiation	Yin et al. (2016)
Ctsk + Scx + TSPCs	Ctsk, Scx	Differentiation into chondrocytes and osteoblasts	Feng et al. (2020)
Cd9+Cd271+ TSPCs	Cd9, Cd271	Secretion of nerve growth factors	Fan et al. (2022)
Gli1+ TSPCs	Gli1	High clonogenicity and multi-lineage differentiation capacity	Fang et al. (2022)
CD26^+^ TSPCs	CD26	Tendon healing and ectopic ossification	Chen et al. (2025)
TDSC-0	AKR1C1, CFD	Inflammatory responses	Guo et al. (2023)
TDSC-1	STC2, HMGA1	Cell migration	
TDSC-2	SLIT3, LUM	Abnormal ECM deposition	
TDSC-3	CENPF, MKI67	Cell proliferation	
TDSC-4	MMP11, FABP5	Inflammatory microenvironment	
TDSC-5	ADIRF, CRABP2	Lipid deposition	
TDSC-6	MXRA5	Tissue repair	
TDSC-7	Low PRDX2, high MALAT1, MEG3	Inhibition of migration; inflammation	

The tubulin polymerization-promoting protein family member 3 (Tppp3), a marker of musculoskeletal development, is specifically expressed in tendon sheaths and paratenon tissues (Goto et al., 2025). Harvey et al. used single-cell sequencing to identify a Tppp3+ Pdgfra + subpopulation with stem cell properties: these cells remain quiescent (Ki67+ <5%) under homeostasis but migrate to injury sites via PDGF signaling post-trauma, differentiating into Scx + tenocytes (Harvey et al., 2019). Goto et al. further demonstrated that activation of the PI3K-Akt signaling pathways in Tppp3+ cells is crucial for tendon repair (Goto et al., 2025). Unlike classical TSPCs, Tppp3+ Pdgfra + cells express high CD34 and minimal Scx (Yin et al., 2016). Some studies also connect Tppp3+ cells to trauma-induced heterotopic ossification (Yea et al., 2023). Pseudotime analysis indicates that Tppp3+ progenitors accumulate early post-injury and then upregulate osteogenic (e.g., Runx2) and tenogenic (Scx, Tnmd) markers, indicating a bifurcation into osteochondrogenic or tenogenic lineages, with potential involvement in ectopic bone formation (Yea et al., 2023). Thus, Tppp3+ progenitors may serve dual roles as repair precursors and contributors to pathological differentiation, though regulatory mechanisms remain unclear.

Recent work has identifies a NESTIN-high TSPC subset critical for tendon repair (Yin et al., 2016). This subpopulation displays enhanced cell cycle activity, particularly in injured tendons (Still et al., 2021). Linking these cells to perivascular niches, Yin et al. demonstrated that they possess superior self-renewal and tenogenic differentiation capacities. Notch signaling regulates these capacities, preventing non-tenogenic lineage commitment and ensuring proper collagen synthesis (Ahn, 2024; Chong et al., 2025).

Additional functional TSPC subsets contribute uniquely to tendon pathology. Feng et al. discovered a Ctsk-Cre + Scx + TSPC subset with robust self-renewal and osteochondrogenic differentiation via Hedgehog (Hh) signaling, implicated in ectopic ossification (Feng et al., 2020). Fan et al. identified Cd9+ Cd271+ TSPCs that secrete neurotrophic factors and become active during the neonatal-to-adult tendon transition (Fan et al., 2022). Some progenitors, such as Gli1+ cells, exhibit clonogenicity and multipotency. They potentially serve as stem cells in regeneration (Fang et al., 2022). Chris Still et al. characterized two mechanoresponsive TSPC subtypes. The first, mrTPCs, are enriched in healthy tendons, expressing mitochondrial genes [MT-ND1/ND4/COX1] and stress-response genes (HSPA1A). These cells enhance energy metabolism in response to mechanical load. The other subtype, piTPCs, represents pro-inflammatory TSPCs that express IL8, CXCL1, and IL6. They recruit immune cells through paracrine signaling (Feng et al., 2020). Resolved TSPC subpopulations exhibit distinct reparative or pathogenic roles (Table 2). For example, CD26^+^ TSPCs are known to promote ectopic ossification. In contrast, Nestin + TSPCs suppress non-tenogenic differentiation via Notch signaling (Yin Z. et al., 2016; Chen S. et al., 2025).

Emerging evidence also suggests alternative TSPC origins. Scx-negative SMA + cells initially reside in the retinaculum and periosteum. After injury, these cells migrate to tendons and differentiate into tenocytes. This implies that paratenon/periosteal tissues may serve as potential reservoirs for TSPC. However, their origins and functions require further validation (Huang et al., 2021). This may represent an additional source of differentiation for tendon repair.

##### 3.1.1.2 Tendon cells

Tendon cells make up most of the cells in tendon tissue and are responsible for building and changing the material around them (Kannus, 2000; Mimpen et al., 2024). Recent studies examining individual cells have found that tendon cell types differ significantly from one another, particularly during the healing process following injury.

The Huang team identified six functional subtypes of cells in a tendon injury model: proinflammatory tenocytes (highly expressing Cxcl5, Cxcl2, and Ccl7), proliferating tenocytes (enriched with Mki67 and Top2a), myofibroblast tenocytes (specifically expressing Sparcl1, Cilp, and Col1a1), signaling tenocytes (highly expressing Cxcl12, Sfrp4, and Gdf10), osteogenic tenocytes (upregulated in Ctsk and Acan), and mature tenocytes. Despite sharing tendon marker genes (Scx, Fmod, Tnmd, Thb), these subtypes exhibit dynamic abundance shifts across repair stages due to divergent functional gene expression profiles, which correlate closely with repair outcomes (Huang Z. et al., 2025).

The Mimpen study expanded the classification of tendon cells. In normal tendons, FBLN1hi cells are high in FBLN1, NOX4, and CILP, and are involved in ECM regulation. ABCA10hi,enriched with ABCA10, CNTN4, and C6, mediate cell adhesion. NR4A1hi express NR4A1, NR4A3, and NAMPT, and may respond to chemical stimuli. Injured tendons, new subtypes emerge: ADAM12hi cells are enriched in COL3A1 and TNC as well as driving fibrosis, whereas aberrant proliferative subtypes express DIAPH3 and TOP2A and promote pathological hyperplasia (Mimpen et al., 2025).

Alternatively, the Micheli team identified three heterogeneous subtypes in healthy mouse tendons. Tendon fibroblasts 1 express osteopontin Spp1 for injury response Tendon fibroblasts are high in dermatopontin Dpt, which stabilizes collagen. Junctional fibroblasts specifically express Col22a1, potentially maintaining tendon-muscle junction integrity (De Micheli et al., 2020). Furthermore, Kendal et al. highlighted pathological subtypes. PTX3+ tenocytes express inflammatory genes CXCL1/6/8 and PDPN, and regulate inflammation. TPPP3/PRG4+ tenocytes express chondrogenic genes, such as COMP, which may drive ectopic ossification and disrupt intrinsic repair (Kendal et al., 2020). These findings suggest that dysfunctional tendon cell subtypes contribute to pathological processes like heterotopic ossification.

The current tendon cell classification methodology lacks standardized criteria, as evidenced by the diverse results in the aforementioned studies. Single-cell studies delineate tenocyte subtypes with divergent functions (Table 3): ADAM12hi tenocytes, which drive fibrosis via COL3A1/TNC overexpression; PTX3+ subtypes, which amplify inflammation through CXCL chemokines—both subtypes are implicated in failed healing (Kendal A. R. et al., 2020; Huang Z. et al., 2025; Mimpen J. Y. et al., 2025) Future studies must establish unified clustering frameworks (Steffen et al., 2023; Sakai and Kumagai, 2025). The Sakai team proposed a functional classification to offer a unified approach:

**TABLE 3:** Tenocyte subpopulations.

Researcher	Cluster name	Unique markers	Function	References
Huang et al.	Proinflammatory Tenocyte	Cxcl5, Cxcl2, Ccl7	Inflammatory response	Huang Z. et al. (2025)
	Proliferating Tenocyte	Mki67, Top2a	Cell proliferation	
	Myofibroblast Tenocyte	Sparcl1, Cilp, Col1a1	-	
	Signaling Tenocyte	Cxcl12, Sfrp4, Gdf10	Cell differentiation	
	Osteogenic Tenocyte	Ctsk, Acan	Ectopic ossification	
	Tenocyte	Scx, Fmod, Tnmd, Thbs (no unique markers)	Tendon healing	
Mimpen et al.	FBLN1hi	FBLN1, NOX4, CILP	ECM dynamics	Mimpen et al. (2025)
	ABCA10hi	ABCA10, CNTN4, C6	Cell adhesion	
	NR4A1hi	NR4A1, NR4A3, NAMPT	(Response to chemical stimuli)	
	ADAM12hi	COL3A1, TNC	Scar formation and low-quality repair post-injury	
	Hyperproliferative	DIAPH3, TOP2A	Pathological hyperplasia	
Micheli et al.	Tendon Fibroblasts 1	Col1a1, Spp1	Injury response	De Micheli et al. (2020)
	Tendon Fibroblasts 2	Col1a1, Dpt	Structural stabilization of collagen	
	Junctional Fibroblasts	Col1a1, Col22a1	Tendon-muscle junction integrity	
Yan et al.	PROCR + Fibroblasts	Casp3, Bax, PROCR	Release of calcified apoptotic vesicles to drive ectopic ossification	Lin P. et al. (2024)
Fu et al.	TC1 (Resident Fibroblasts)	MDK, PDGFRB, FBLN2, COL1A1	Resident fibroblasts; tendon growth/differentiation	Fu et al. (2023)
	TC2	MEG3, EGR1, DCN, COL1A2, FBLN1	Tenocyte proliferation and ECM synthesis	
	TC3	PLA2G2A, SCARA5, PLPP3, GPNMB	Defense-related homeostatic fibroblasts	
	TC4	MYOC, IGFBP6, THBS4, CILP, CHAD	Localization at tendon-bone insertion	
	TC5	HAS1, PRG4	Endochondral ossification	
	TC6	SAA1, PTGFR, STEAP1, RARRES1	Inflammation	
	TC7	TPPP3, COL3A1, COL5A1, DPT	Scar-mediated healing	
Yoshimoto et al.	Mature Tenocytes	Tnmd, Col1a2, Scx, Mkx	Tendon growth/differentiation	Yoshimoto et al. (2022)
	Differentiating Tenocytes	Scx, Tgfb2	Transitional state between progenitors and mature tenocytes	
Kendal et al.	Tenocyte A	PTX3, FBN1, MFAP5, CXCL1, CXCL 6, CXCL 8, PDPN	Inflammatory response	Kendal et al. (2020)
	Tenocyte B	Krt7, Scx, Fbn1, Mfap5, Vcan Emilin1	Production of tendon microfibrils	
	Tenocyte C	Itga7, Tagln, Myl9, Acta2, Rgs5	Angiogenesis	
	Tenocyte D	Apod, Col3a1, Cxcl14 Gsn, Lum, Dcn, Ly6e, Pdgfra	Fibrosis	
	Tenocyte E	COMP, FMOD, CILP	Production of reparative matrix, related to ectopic ossification	
Steffen et al.	Tendon fibroblasts 1	Col1a1, Fmod, Comp, Chad	Type I collagen production	Steffen et al. (2023)
	Tendon fibroblasts 2	Apoe, Col3a1, Cfd, Tmsb4x, Gsn	Production of circumferential collagen	
Ackerman et al.	Synthetic Tenocyte	Tnmd, Col1a1, Fmod	ECM synthesis	Ackerman et al. (2022)
	Native Tenocyte	Coch, Chad, Car3	Resemble normal tenocytes	
	Reactive Tenocyte	Mmp13, Lox, Fbln2	Adhesion, migration, and proliferation	
	Fibrotic Tenocyte	Col3a1, Postn, Thbs3	Fibrosis	
	Inflammatory Tenocyte	Saa3, S100a8, S100a9, Lcn2	Inflammation	

ECM-synthetic (Col1a1, Fmod), involved in extracellular matrix production; ECM-remodeling (late repair), associated with matrix reorganization; inflammation-modulatory (tissue clearance), mediating immune responses; and fibrogenic (scar-forming) subtypes, responsible for fibrosis (Sakai and Kumagai, 2025). Notably, this system excludes osteogenic subtypes (expressing Ctsk, Acan, COMP), which may critically underlie repair failure (Kendal et al., 2020; Huang Z. et al., 2025). A consensus classification system is urgently needed to systematically decode regulatory networks in physiological repair and pathological progression.

##### 3.1.1.3 Differentiation trajectories of TSPCs and their dynamic roles in injury

As described above, the differentiation trajectories of TSPCs and their resultant tendon cell subtypes have a significant influence on tendon healing outcomes. Understanding their dynamic changes during repair provides critical insights for therapeutic interventions.

Huang et al. demonstrated that multiple tendon cell subtypes originate from progenitor cells (Dyment et al., 2013; Kan et al., 2024; Huang Z. et al., 2025). Building on these findings, pseudotime trajectory analysis revealed four major differentiation branches: 1) Direct differentiation into proliferating tenocytes; 2) Generation of signaling tenocytes; 3) Sequential differentiation into signaling tenocytes followed by osteogenic tenocytes; 4) Differentiation into myofibroblast tenocytes, which may further mature into tenocytes. Notably, in injured tendons, differentiation often stalls at the myofibroblast tenocyte stage, leading to fibrotic tissue formation rather than functional maturation (Huang Z. et al., 2025). In a related study, Yoshimoto et al. validated a maturation trajectory (Aldh1a2+ progenitors → Scx + cells → Tnmd + tenocytes) using single-cell sequencing (Yoshimoto et al., 2022).

Huang’s work further linked differentiation trajectories to repair timelines. In healthy tendons, progenitor cells (e.g., Tppp3+, Sca-1+ subtypes) are abundant. During the early stages of injury (Day 1), progenitors decline. They differentiate into either proliferating tenocytes (expressing Mki67, Top2a) to fill defects or proinflammatory tenocytes (high in Cxcl5, Ccl7) to amplify inflammation (Huang Z. et al., 2025). By mid-repair (Day 7), proliferating tenocytes have become the dominate cell type. Progenitors also generate signaling tenocytes (high in Col1a1, Thbs4). Under Hedgehog activation or oxidative stress, progenitors may aberrantly differentiate into osteogenic tenocytes (expressing Ctsk and Acan) or myofibroblast tenocytes (expressing α-SMA and Sparcl1) (Feng et al., 2020; Huang Z. et al., 2025). In late repair (Days 14–28), proliferating tenocytes diminish. Successful healing restores mature tenocyte proportions and near-normal ECM architecture (thick collagen fibers), with upregulated genes enriched in ECM synthesis, adhesion, cytokine production, and metabolism (Huang Z. et al., 2025). Conversely, poor healing or heterotopic ossification involves persistent myofibroblast tenocytes secreting scar collagen (COL3A1) or TSPC differentiating into osteogenic tenocytes (expressing Runx2 andSPP1) with calcific vesicle deposition (Fu et al., 2023; Kan et al., 2024).

Fu et al. demonstrated that TSPCs in healthy tendons differentiate into mature tenocytes and attach to maintain function (Fu et al., 2023). In injured tendons, TSPCs generate pathological repair-associated fibroblasts, chondrocytes, and osteocytes (Fu et al., 2023). Notably, progenitors cells expressing tubulin polymerization-promoting protein family member 3 (Tppp3+) rapidly multiply after injury but are more likely to become bone- or cartilage-forming cells (osteogenic/chondrogenic lineages) by increasing the activity of the gene Runx2. This shift helps explain the frequent occurrence of abnormal bone formation (ectopic ossification) and tissue scarring (fibrosis) (Fu et al., 2023; Yea et al., 2023). PDGF-AA stimulation promotes Tppp3+Pdgfra + progenitors to adopt tenogenic fates, while Pdgfra inactivation disrupts regeneration (Harvey et al., 2019).

These findings underscore that healing depends on the differentiation paths of TSPC. Thus, changing the fate of these cells may help regenerative repair (Huang Z. et al., 2025). Existing studies explain adverse outcomes through mechanisms specific to cell lineages. However, comprehensive pseudotime analyses (ordering cells along developmental pathways) are rare. This leaves gaps in understanding tendon cell origins and hierarchies. Future research must clarify cell lineages to advance therapies.

#### 3.1.2 Immune cells

##### 3.1.2.1 Macrophages

Macrophages play a central regulatory role in tendon healing. In particular, their functional diversity and phenotypic plasticity make them key effector cells that coordinate the balance between inflammatory responses, tissue remodeling, and regeneration (Crosio and Huang, 2022; Li et al., 2025b; Nichols et al., 2025).

In traditional studies, macrophages are classified into two main phenotypes: pro-inflammatory M1 and pro-repair M2 phenotypes (Zhou M. et al., 2025). Yan et al. observed dynamic phenotypic switching of macrophages during tendon injury, where pro-inflammatory M1 macrophages (Nos2+, Il1b+) dominate in the early phase (1-week post-injury) and transition to pro-repair M2 macrophages (Arg1+, Vegfa+) by week 3. Pseudotime analysis confirmed a continuous differentiation trajectory from M1 to M2 phenotypes (Lin P. et al., 2024). Kan et al. further subdivided M2 macrophages into injury-recruited clusters (Mrc1+, Tgfb1+) and tissue-resident clusters (Cd163+, Retnla+), which regulate MSC differentiation and tissue homeostasis, respectively (Kan et al., 2024). While some studies avoid the M1/M2 nomenclature, most categorize macrophage subtypes based on their inflammatory profiles.

In a needle-puncture-induced tendon injury model, researchers identified several macrophages subtypes: reactive macrophages, regulatory macrophages 1 and 2, and teno-macrophages (Huang Z. et al., 2025). Reactive macrophages resemble the pro-inflammatory M1 phenotype and initiate early inflammation (Ackerman et al., 2021). Regulatory macrophage 1 expresses high levels of immunomodulatory genes such as Tlr2 and IL-10. Regulatory macrophage 2 influences macrophage behavior via chemokines like Mrc1, Ccl7, and Ccl8; both regulatory subtypes are more abundant in later injury stages. Teno-macrophages co-express monocyte/macrophage markers (Cd14, Cd68) and tenogenic markers (Tppp3, Ecm1). This suggests that these cells are tissue-resident and may regulate fibrosis through Fabp5 and Trem2 (Qiu et al., 2017; Huang Z. et al., 2025). Notably, researchers also identified myeloid-derived tenogenic cells expressing the myeloid marker F4/80 and the tendon marker Col1, suggesting that some tendon cells may derive from the myeloid lineage (Huang Z. et al., 2025).

In quadriceps tendon studies, macrophages in healthy and injured tendons were classified into MERTKhi LYVE1hi, MERTKhi LYVE1lo, and MERTKlo PTPRGhi subsets. Macrophage heterogeneity, meaning the diversity of macrophage types, is now resolvable (Table 4). Healthy tendons predominantly contain LYVE1hi macrophages, whereas injured tendons show increased LYVE1lo populations expressing pro-inflammatory chemokines CXCL2/3/8. The PTPRGhi subset uniquely expresses the noncoding RNA MIR99AHG, potentially modulating macrophage phenotype via IL-4/IL-13 signaling (Mimpen et al., 2025).

**TABLE 4:** Immune cell subpopulations.

Cell type	Cluster name	Unique markers	Function	References
Macrophage	M1 Macrophage	Nos2, Il1b	Inflammation and stem cell proliferation	Kan et al. (2024), Lin P. et al. (2024)
	M2 Macrophage	Arg1, Vegfa	Repair and differentiation; (ectopic ossification)	
Macrophage	Injury-recruited M2 Macrophage	Mrc1, Tgfb1	Stem cell differentiation	Kan et al. (2024)
	Tissue-resident M2 Macrophage	Cd163, Retnla	Tissue homeostasis	
Macrophage	Reactive Macrophage	Il1b	Early inflammation	Huang Z. et al. (2025)
	Regulatory Macrophage 1	Tlr2, Tlr26, Il10, Il27	Suppression of inflammation	
	Regulatory Macrophage 2	Mrc1, Ccl7, Ccl8, Ccl29	Suppression of inflammation	
	Teno-macrophage	Cd14, Cd5, Cd68, Tppp3, Ecm1	Repair, reduction of inflammation, and fibrosis	
Macrophage	MERTKhi LYVE1hi Macrophage	—	Tissue-residency in healthy tendon	Mimpen et al. (2025)
	MERTKhi LYVE1lo Macrophage	CSTB, TPTRG1, HMOX1, CXCL2, CXCL3, CXCL8	Inflammation	
	MERTKlo PTPRGhi Macrophage	MIR99AHG, IL-4, IL-13	Macrophage phenotype switching	
T Cell	CD4-/CD8- γδ T Cells	Trdc, Tcrg	—	Arvind et al. (2025)
	NK-like T Cells	Nkg7, Cd8b1, Klrd1, Klrc1, Klrk1	—	
	CD8^+^ Effector T Cells	Cd8b1, Cd8a	(ectopic ossification)	
	Neonatal Treg	Areg, Tgfb1, Il1rl1, Il4ra	Suppression of inflammation and acceleration of repair	
	Adult Treg	Ifng, Tnf	Inflammation and suppression of repair	
T Cell	CD4^+^ T Cells	Cd3e, Cd4	(ectopic ossification)	Kan et al. (2024)
	CD8^+^ T Cells	Cd3e, Cd8a	(ectopic ossification)	
	NKT Cells	Klrb1c, Cd3e	—	
	γδ T Cells	Cd3e (low Klrb1c, Cd4, Cd8a)	—	

Chronic tendon injury studies reveal a shift from repair-oriented macrophages (LYVE1+, APP+) to pro-inflammatory macrophage phenotypes, accompanied by MIF/CD74 pathway activation and increased cycling macrophages (Akbar et al., 2021). Muscat et al. demonstrated the critical role of CCR2+ macrophages in late-stage repair; their depletion reduces myofibroblasts and impairs functional recovery. These findings systematically elucidate the spatiotemporal regulatory network of macrophage subsets in tendon repair (De Micheli et al., 2020).

##### 3.1.2.2 T Cells

T cell subsets display substantial heterogeneity and functional specificity during tendon regeneration. Arvind et al. used single-cell sequencing to identify six distinct T cell subtypes in neonatal and adult tendon healing: γδ T cells (Trdc+, Tcrg+, Trdc1+), NK-like T cells (Nkg7+, Cd8b1+, Klrd1+, Klrc1+, Klrk1+), CD8^+^ effector T cells (Cd8b1+, Cd8a+), and two Regulatory T cell (Treg) subsets (CD4+/Foxp3+). Critically, neonatal Tregs express high levels of tissue repair genes (Areg, Tgfb1) and type 2 immune receptors (Il1rl1, Il4ra), whereas adult Tregs remain quiescent (Arvind et al., 2025). Neonatal Tregs dynamically modulate the inflammatory microenvironment through the IL-33/ST2 axis (encoded by IL1rl1), clearing transient IL-33 elevation via receptor-mediated uptake. This promotes polarization of anti-inflammatory macrophage (Ly6Clo), restores TGF-β/SMAD signaling, and enables effective regeneration. Conversely, adult Tregs upregulate pro-inflammatory genes (Ifng, Tnf) and lack repair functions, leading to IL-33 accumulation, Ly6Chi macrophage-driven chronic inflammation, and collagen disorganization. These findings highlight the key role of T cell-mediated modulation in the regenerative microenvironment (Arvind et al., 2025). Building on this, Kan et al. found that persistent CD4^+^ Th1 and CD8^+^ T cells in injured tendons secrete IFNγ, activating the PI3K/AKT pathway to drive MSC chondrogenesis, which may potentially result in ectopic ossification-an effect that is reduced by T cell depletion (Kan et al., 2024). Taken together, these studies systematically elucidate the spatiotemporal regulatory mechanisms by which T cell subsets influence tissue repair outcomes.

### 3.2 Intercellular interactions and tendon prognosis

Single-cell sequencing technologies have revealed how intercellular interactions shape tendon healing. Tendon lineage cells, immune cells, and other cell types​​ form a tripartite regulatory network that is crucial for achieving optimal healing outcomes. Tendon lineage cells build the tendon matrix, modulate inflammation, and promote angiogenesis. Immune cells regulate the microenvironment, affecting both the states of tenocytes and endothelial cells. Endothelial cells not only supply nutrients for regeneration but also modulate the functions of tendons and immune cells. Despite these valid influences on recovery, most current research centers on pathological crosstalk in injury.

#### 3.2.1 Regulatory networks between tendon lineage cells and immune cells

In the early post-injury phase, pro-inflammatory macrophages (TNF+, IL1α+) activate quiescent TSPCs via TNF-α and IL-1α secretion. This engages TNFRSF1B receptors on TSPCs, leading to STAT3 signaling (threefold increase in phosphorylation levels) and promoting TSPC proliferation. Macrophage depletion via clodronate liposomes reduces MSC populations by 60% (Kan et al., 2024). Resident macrophages, identified in adult tendons by scRNA-seq, help maintain tissue homeostasis. In CCR2 knockout models, the absence of macrophages correlates with 60% fewer tenocytes and impaired late-phase healing (Shen et al., 2025). Proinflammatory tenocyte subsets sustain inflammatory microenvironments through IFN-mediated signaling and IL-1-driven NF-κB activation (Kendal et al., 2020).

During tendon injury repair, macrophages gradually transition toward a pro-reparative phenotype. Mrc1+ macrophages and other tissue-reparative M2-type macrophages exhibit increased abundance starting at day 3 post-injury, indicating their active engagement in repair initiation (Kan et al., 2024). These pro-reparative macrophages exert dual regulatory effects on tendon healing. On one hand, they mitigate local inflammatory responses to establish a favorable healing microenvironment, releasing TGF-β and other signaling molecules that activate SMAD pathways to drive TPSC differentiation and tenocyte regeneration. On the other hand, pro-reparative macrophages regulate osteogenic differentiation of TSPCs through two key mechanisms. First, they secrete galectin-9 (LGALS9), which binds to CD44 on TSPCs, activating PI3K/AKT signaling pathway to promote SOX9+ chondroprogenitor formation (Kan et al., 2024). Second, they produce oncostatin M, which interacts with specific receptors on TSPCs, activating RUNX2 transcription to drive osteogenic differentiation. Neutralization of pro-reparative macrophages reduces ectopic bone formation by 65% (Kan et al., 2024). Notably, studies have shown that during late tendon repair, macrophages undergo phenotypic switching from LYVE1+ tissue-reparative to MIF/CD74+ pro-inflammatory phenotypes. This phenotypic shift perpetuate NF-κB pathway activation and drives abnormal TSPC differentiation toward chondro/osteogenic lineages rather than tenocytes (Akbar et al., 2021). Finally, in later repair phases, tenocytes themselves contribute to the resolution stage by producing anti-inflammatory factors such as IL-10 and TGF-β, which promote tissue repair while suppressing inflammation and facilitating remodeling (Huang Z. et al., 2025).

**FIGURE 2:**
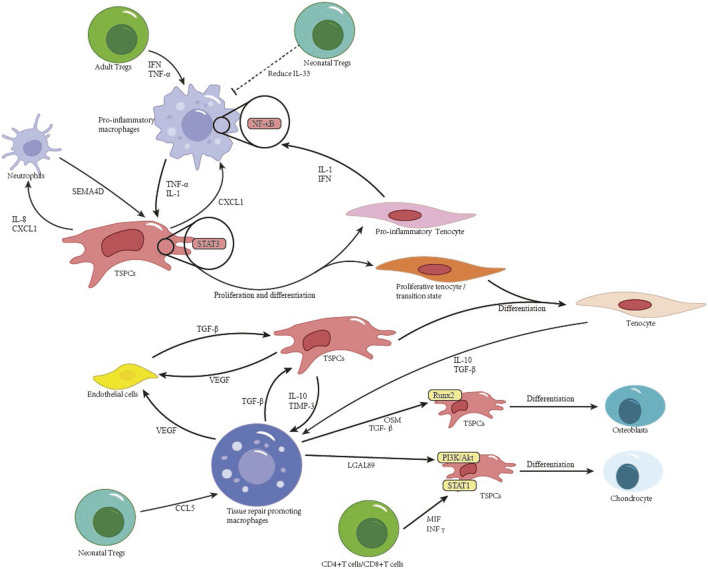
Cellular interactions and stem cell differentiation in tendon healing.

T cells play a critical role in regulating tendon lineage differentiation. Tregs in neonatal injury models highly express anti-inflammatory genes (e.g., Il10, Tgfb1), steering early immune microenvironments toward regeneration (Arvind et al., 2025). However, late-phase T cell activity correlates with heterotopic ossification: CD4^+^ Th1 cells secrete IFN-γ, which activates STAT1 signaling in TSPCs, thereby upregulating SOX9 for chondrogenesis (Kan et al., 2024). CD8^+^ T cells secrete MIF to engage CD74 receptors on TSPCs, inducing NF-κB activation (threefold increase in p65 nuclear translocation) and RUNX2/BSP expression. CD8^+^ T cell depletion reduces ectopic bone volume by 40% at day 14 (Akbar et al., 2021). The tripartite crosstalk among tendon lineage cells, immune subsets, and endothelial cells dictates healing outcomes. Figure 2 synthesizes these interactions, emphasizing how macrophage-derived TNF-α activates TSPC proliferation via STAT3, while aberrant T cell signaling (e.g., IFN-γ) drives pathological osteogenesis (Kan C. et al., 2024).

#### 3.2.2 Coordinated remodeling mechanisms between tendon lineage and vascular cells

Pathological tendon remodeling involves synergistic interactions between cellular subsets via defined signaling axes. Specifically, ADAM12hi tenocyte subsets interact with vascular endothelial cells through the TGF-β1-TGFβR2/Smad3 axis, upregulating COL1A1 and COL3A1 mRNA to increase collagen density in injury cores (Ng et al., 2024). CXCL12+ endothelial cell subsets, in spatial proximity to macrophages, activate the SPP1-PTGER4/TGFB1-TGFβR1 axis, which in turn increase SOX9/RUNX2 expression in TSPCs, thus promoting ectopic ossification (Fu et al., 2023). PDGFRβ+/BMP2+ pericytes contribute via dual pathways: (1) BMP2-ACVR1 signaling drives SMAD1/5 phosphorylation in TSPCs, initiating RUNX2/SP7-dependent osteogenesis; (2) JAG1-NOTCH3 signaling converts pericytes into α-SMA + myofibroblasts, exacerbating fibrosis (Akbar et al., 2021). Additionally, vascular endothelial cell-derived CXCL12 mediates TSPC chemotaxis via CXCR4, highlighting endothelial regulation of stem cell recruitment (Kendal et al., 2020). These findings systematically delineate the hierarchical regulatory networks driving pathological remodeling, offering multidimensional therapeutic targets. Figure 2 synthesizes these cellular interactions, highlighting how tenocyte-immune crosstalk dictates healing outcomes. Healing of the Enthesis.

The healing process is harder at the tendon-bone connection (enthesis) than in the tendon alone. The is because the enthesis is made up of four layers: tendon, soft cartilage, hard cartilage, and bone. (Ding Z. et al., 2025; Matsui and Tanaka, 2025). These zones exhibit sequential cellular differentiation from tendon fibroblasts to chondrocytes, mineralized chondrocytes, and osteoblasts/osteocytes (Chen Y. et al., 2021). This structural complexity differs significantly from simple tendon healing and is critical for stress distribution and energy transfer. Notably, the fibrocartilage zone absorbs concentrated stresses, protecting the bone from excessive shear forces (Jiang F. et al., 2024; Bassil et al., 2025; Sensini et al., 2025). Such an ordered structure enables efficient stress transmission from tendon to bone (Rossetti et al., 2017). However, current therapeutic approaches fail to achieve anatomical reconstruction of this multilayered architecture after enthesis injury (Huang M. et al., 2025). Although surgical techniques restore mechanical continuity between tendon and bone, they cannot regenerate the native tissue gradient, compromising stress distribution and increasing vulnerability to re-rupture (Patel et al., 2016).

The current understanding of enthesis healing follows three phase model, inflammatory, proliferative, and remodeling phases (Dai et al., 2025; Ding Z. et al., 2025). Initially, localized cytokine and growth factor release recruits neutrophils, monocytes, and macrophages to clear debris and initiate repair (Hegedus et al., 2010). Next, the proliferative phase involves fibroblast infiltration, collagen deposition, and stem cell differentiation (Hegedus et al., 2010). Finally, newly synthesized ECM integrates with bone, establishing collagen continuity between the tendon graft and bone (Deehan and Cawston, 2005; Hegedus et al., 2010).

However, this approach is significantly simpler than the complex interactions between cells and cell groups during tendon healing. Despite limited research on enthesis healing at the single-cell level. Research on enthesis healing at the single-cell level remains limited (Fang et al., 2025). Emerging studies utilizing single-cell sequencing have begun to resolve high-resolution cellular dynamics during enthesis healing or development, but this field remains in its infancy. This section summarizes current findings from single-cell sequencing-based investigations.

### 3.3 Cellular types at the enthesis

Current research on the cellular composition at the enthesis primarily focuses on two origins: mesenchymal-derived cells and myeloid-derived cells (Xu Y. et al., 2025). Traditional studies propose three distinct progenitor populations during enthesis development: tendon midsubstance progenitors, enthesis progenitors, and primary cartilage progenitors (Dyment et al., 2015). The morphogenesis of the enthesis involves the transformation of enthesis progenitors into fibrocartilage, forming an unmineralized cartilaginous attachment unit that undergoes postnatal mineralization via endochondral ossification (Zhang T. et al., 2023).

Recent single-cell sequencing studies by Gao et al.identified mesenchymal-derived cell types at the enthesis healing, including mesenchymal stem cells (Cebpd, Ly6a, Pdgfra), TSPCs (Tnc, Scx, Tagln), tenocytes (Tnmd, Fmod, Thbs4), fibroblasts (Mfap4, Mest, Fth1), chondrocytes (Col2a1, Sox9, Col10a1), adipocytes (Lpl, Cyp7b1, Creb3l3), myofibroblasts (Ppp1r14a, Myh11, Parm1), and osteocytes (Bglap, Col1a1, Dmp1). Despite this comprehensive identification, the study used only conventional cell categories and did not further subtype mesenchymal-derived populations (Gao et al., 2025).

Fang et al. refined the classification of mesenchymal-derived cells into six subtypes: enthesis progenitors (Gli1, Ly6a, Cd34, Cd44, Pdgfra); pre-enthesoblasts (low Ly6a/Cd34 and high Sox9), representing a transitional state; enthesoblasts (Scx, Tnmd, Sox9, Acan, Col1a1), mediating matrix deposition in the tendon-bone transition zone; mineralizing chondrocytes (Sox9, Acan, Col2a1, Alpl, Spp1, Ibsp), driving interface mineralization to establish an ossification gradient; tenoblasts/tendon sheath cells (Scx, Tnmd, Col1a1, Bglap), maintaining tendon matrix synthesis and remodeling; and osteocytes (Nfatc1, Bglap, Spp1, Dmp1), regulating bone matrix mineralization and homeostasis (Fang et al., 2022). Fang et al. traced the differentiation trajectory of Gli1+ progenitors through pre-enthesoblasts, enthesoblasts, and mineralizing chondrocytes, highlighting their role in enthesis development and regeneration (Fang et al., 2022). This aligns with the results of Zhang et al., who confirmed that enthesis fibrocartilage originates from enthesis-specific progenitors rather than tenocytes (Zhang T. et al., 2023). Similarly, Zhang et al. classified enthesis cells as chondrocytes, tenoblasts, mesenchymal progenitors, osteoblasts, and enthesoblasts. Fu et al. identified two unique enthesis cell populations: bone-adjacent cells expressing cartilage lubricants HAS1 and PRG4 (linked to endochondral ossification), and tendon-proximal cells expressing fibroblastic markers MYOC and IGFBP6 (Fu et al., 2023). However, apparent discrepancies exist: lineage-tracing studies suggest that the enthesis’s multilayered architecture derives exclusively from tendon-side progenitors, rather than from bone or cartilage lineages, suggesting context-dependent interpretations (Wang Z. et al., 2023; Pugliese et al., 2024).

Although immune-inflammatory responses are key to enthesis healing (Fujii et al., 2022; Romereim et al., 2025), single-cell immune cell profiling remains limited. To address this, Gao et al. grouped macrophages into three subtypes: pro-inflammatory macrophages (Nlrp3, Il1b, Il6, Ptgs2, Ly6c2), which activate the NLRP3 inflammasome and secrete interleukin-1β (IL-1β)/IL-6 to inhibit regeneration; anti-inflammatory macrophages (Ccl8, C1qa, Mrc1, Arg1), which promote inflammation resolution via IL-10/IL-13 to support stem cell differentiation; and osteoclasts (Acp5, Atp6v0d2, Ctsk, Mmp9), which mediate bone resorption and remodeling (Gao et al., 2025). Additionally, while neutrophils and T cells were identified, they were not classified into specific in these studies.

### 3.4 Intercellular interactions in enthesis healing

During the early post-injury phase, ATP released from damaged tissues binds to the P2X7 receptor on macrophages, activating pro-inflammatory macrophages and triggering the assembly of the NLRP3 inflammasome. This process, in turn, drives excessive IL-1β secretion, which suppresses MSC migration and differentiation via IL-1β/IL-1R signaling pathway (Gao et al., 2025). Meanwhile, neutrophils recruit endothelial cells through CXCL12-CXCR4 chemokine interactions, thereby promoting aberrant angiogenesis (Fu et al., 2023).

As healing progresses, anti-inflammatory macrophages increase in number and secrete IL-10 and IL-13. Specifically, IL-10 activates TGF-β signaling in enthesis progenitors, thereby directing their differentiation toward chondrogenic lineages (Zhang T. et al., 2023). In addition, Anti-inflammatory macrophages release docosatrienoic acid, a polyunsaturated fatty acid that activates the PI3K/Akt pathway to promote the proliferation and regeneration of progenitor cells (Gao et al., 2025).

During the mid-to-late stages of healing, mesenchymal progenitors receive pro-differentiation signals through FGF2-FGFR2 and BMP2-BMPR2 interactions. These signals sustain chondrogenic differentiation and stimulate cartilage-specific ECM synthesis (Zhang T. et al., 2023; Gao et al., 2025). Simultaneously, IL-13 is secreted by anti-inflammatory macrophages. This activates the STAT6 pathway and promotes cartilage matrix mineralization (Gao et al., 2025).

However, despite these advances in understanding healing mechanisms, current single-cell studies in bone-tendon interface enthesis healing remain limited. Consequently, there is a need for further research to comprehensively map cellular crosstalk and regulatory networks in this specialized microenvironment.

### 3.5 Perspective: reconciling lineage origins of the enthesis

The contradiction between tendon-centric and enthesis-specific progenitor origins in fibrocartilage formation stems from differences in methods and experimental contexts. Tendon-origin studies usually investigate embryonic development using lineage tracing of Scx + populations in mice (Wang Z. et al., 2023). In contrast, analyses that support enthesis-specific progenitors often focus on post-injury repair and Gli1+ cells (Zhang T. et al., 2023). Technical limitations also cause confusion. Traditional lineage tracing cannot resolve the transitional cellular states that occur during healing. On the other hand, single-cell RNA sequencing (scRNA-seq) identifies locally activated progenitor phenotypes without clear links to their developmental origins. Finally, ambiguity in the term ‘enthesis progenitor’ adds to the issue. It can refer to either the location at the insertion site or a commitment to fibrocartilage differentiation.

Current multimodal single-cell methods resolve these conflicts by using pseudotime trajectory reconstruction, which demonstrates how tendon-derived progenitors (Tppp3+/Scx+) gain fibrocartilaginous signatures (Sox9+/Acan+) during enthesis maturation (Fang F. et al., 2022; Huang M. et al., 2025). Furthermore, spatial transcriptomics localizes Gli1+ cells to the enthesis zone, confirming their developmental origin from tendon-side niches and revealing their transcriptional specialization as they differentiate (Steffen D. et al., 2023; Fang F. et al., 2025). Collectively, these findings support a unified model: tendon-resident progenitors create enthesis niches during development and later reactivate context-dependent identities during repair, thereby reconciling previously conflicting lineage paradigms.

## 4 Summary and perspectives

In recent years, single-cell sequencing technology has demonstrated unique power in tendon and tendon-bone healing research. This technology offers novel insights into the cellular and molecular mechanisms that drivetissue regeneration and pathological remodeling. Studying tendon and enthesis healing at the single-cell level is a significant advance in understanding tissue repair biology. Current studies have identified key cellular subtypes and their functions in the tendon microenvironment, such as the heterogeneous differentiation trajectories of TSPCs, phenotype-switching patterns of macrophage subsets, and immune-tenocyte interaction networks. However, several challenges remain exist. First, single-cell studies focusing on enthesis healing remain scarce, leaving the spatiotemporal distribution and interaction networks of cellular subtypes across its complex four-layer architecture (tendon-fibrocartilage-mineralized cartilage-bone) unresolved. Second, temporal resolution of healing processes remains incomplete due to limited application of time-series analyses and spatial transcriptomics. Third, cross-species heterogeneity (e.g., gene expression disparities between rodents and humans) may lead to misinterpretation of regulatory pathways, hindering clinical translation. Addressing these challenges requires systematic approaches to integrate and expand existing findings, establishing a unified theoretical framework to comprehensively decipher the biological principles of tendon and enthesis healing.

In this review, our primary focus centered on the heterogeneity and differentiation trajectories of TSPCs. Single-cell sequencing analyses have revealed the differentiation trajectories and refined the subclustering of TSPCs, further underscoring their pivotal roles in tendon healing. Current findings demonstrate substantial heterogeneity within the TSPC population. Even Tppp3+ TSPCs exhibit functional diversity, with their proliferative and differentiation capacities proving critical during tendon or enthesis repair. However, their involvement in ectopic ossification during tendon injury has also been extensively documented (Yea et al., 2023). We propose two plausible explanations. First, Tppp3+ TSPCs may undergo microenvironment-dependent fate specification, where immune-derived signals or matrix stiffness modulates their differentiation. Apparent contradictions regarding enthesis cellular origins—whether tendon-derived or locally specified—reflect contextual and technical distinctions rather than biological contradictions. Integrated scRNA-seq and spatial mapping demonstrate that tendon-side progenitors seed enthesis niches during development and adopt injury-induced transcriptional states, reconciling these paradigms (Zhang T. et al., 2023; Gao H. et al., 2025). Molecularly, the functional heterogeneity and lineage divergence of TSPCs fundamentally reflect competitive activation between osteochondrogenic signals (Runx2 and Sox9) and tenogenic programs (Scx/Tnmd), which likely involves unresolved epigenetic regulatory mechanisms. Consequently, factors such as matrix stiffness, immune microenvironmental shifts, mechanical stimuli, and neural paracrine signaling may critically influence these processes (Yu et al., 2025), though current scRNA-seq studies have yet to explore such mechanisms in depth. Alternatively, Tppp3+ TSPCs might represent a broad cellular category, consistent with prior reports (Kendal et al., 2020; Goto et al., 2025). While Tppp3+ TSPCs constitute a distinct TSPC subset, their classification based on Tppp3 expression does not necessarily imply functional uniformity in proliferation or differentiation. Tppp3, a member of the tubulin polymerization-promoting protein family, is implicated in diverse biological processes, including lactate metabolism (Liu et al., 2024). Its broad tissue distribution and functional roles in pancreatic cancer, neurological disorders, germ cell development, and macrophage polarization have been well-documented (Ding M. et al., 2025; Pang et al., 2025; Ren et al., 2025; Zhu et al., 2025). However, whether Tppp3 truly marks a homogeneous subpopulation remains debatable. Previous scRNA-seq studies emphasize the necessity for iterative subpopulation refinement or reclassification (Ito et al., 2025), suggesting that current Tppp3+ TSPC definitions may require further resolution.

We also focused on the current utilization of single-cell sequencing data in tendon disorders and future technological prospects. Most existing scRNA-seq studies in tendons primarily aim to identify cellular subpopulations and analyze their functions. However, single-cell research in tendon healing remains relatively underdeveloped. This fact must be clearly acknowledged. Emerging technologies warrant greater attention as they may revolutionize current understanding. The integration of scRNA-seq, spatial transcriptomics (Spatial-seq), and proteomics represents a crucial developmental direction. Multi-omics integration enables comprehensive analysis of cellular heterogeneity and spatial localization. Most current studies predominantly rely on gene-level data to infer interactions between cellular subtypes. However, spatial consistency is a prerequisite for meaningful cellular subtypes. However, spatial consistency is necessary for meaningful cellular crosstalk. Spatial transcriptomics is needed to map spatial information (Beck et al., 2025; Chowdary et al., 2025). Furthermore, spatial transcriptomics applications could directly resolve the distribution and functional states of diverse cell types within healing zones. This advancement would significantly enhance our understanding of subpopulation roles and immune microenvironmental dynamics (Fetahu et al., 2023). Moreover, multi-omics integration allows dynamic tracking of molecular events and regulatory mechanisms. A persistent challenge in tendon healing research is deciphering the differentiation trajectories and regulatory mechanisms of tendon-lineage cells. ScRNA-seq, combined with pseudotemporal analysis, enables preliminary reconstruction of differentiation trajectories. Integration with proteomics can validate the activation timing of key pathways through post-translational modifications, such as phosphorylation and glycosylation. This approach precisely resolves signaling pathways, particularly those governing the lineage commitment of TSPCs (Tan et al., 2025). Such multi-omics approaches have already demonstrated substantial success in oncology research (Tseng et al., 2025; Wang S. et al., 2025).

Another critical direction for unraveling the mechanisms of tendon repair involves integrating artificial intelligence (AI) analytical strategies with single-cell RNA sequencing technology. The massive, high-dimensional data generated by scRNA-seq pose significant challenges to conventional analytical approaches. Here, AI intervention emerges as a critical solution. Current exemplary applications of AI in scRNA-seq data analysis include tools such as scANVI (single-cell ANnotation using Variational Inference) and CellChat. scANVI is a deep generative model and core component of the scvi-tools framework (Luecken et al., 2022; Andreatta et al., 2024). It excels in integrating multi-batch single-cell data through semi-supervised learning. scANVI leverages existing scRNA-seq datasets as references to annotate novel sequencing results. It also infers biological states of unlabeled cells using minimal prior cell-type information (Song Y. et al., 2023; Danino et al., 2024). This significantly enhances the accuracy of cellular subpopulation annotation. For instance, in neuroscience, scANVI has proven instrumental in resolving cross-species correspondence of neuronal subpopulations in human, mouse, and macaque studies (Chen X. et al., 2024). For tendon and enthesis research utilizing animal models, scANVI enables in-depth subpopulation characterization. It may also establish standardized cross-study alignment of heterogeneous cell clusters and address noise interference caused by low cell capture rates in tendon injury models. CellChat is another valuable tool for tendon and enthesis regeneration studies. It is a ligand-receptor database-driven cell communication analyzer. It deciphers global regulatory networks of signaling pathways through graph theory and pattern recognition (Dupuis et al., 2025; Yang et al., 2025). This approach is particularly significant for pathophysiological processes involving multicellular interactions. It extensive applications in tumor microenvironment studies have helped to dissect immune cell-tumor cell crosstalk (Guan et al., 2025; Wang H. L. et al., 2025; Zhang et al., 2025). Given the intricate, multicellular, and multi-subpopulation interactions in tendon and enthesis biology, such analyses represent a crucial future direction for scRNA-seq research. However, current tendon studies have yet to extensively employ CellChat or similar tools for systematic interaction analysis (Liu W. et al., 2025). Future applications could elucidate temporal regulatory mechanisms governing TSPCs. They could also identify activated signaling pathways and differentiation trajectories to inform the development of targeted therapies. Furthermore, integrating human scRNA-seq data with deep learning models and generative pre-training frameworks enables the extraction of universal biological principles from vast datasets. Emerging AI methodologies such as GPTCelltype and scGPT demonstrate remarkable capabilities in automated cell subtype annotation (Cui et al., 2024; Hou and Ji, 2024). While scRNA-seq captures static cellular snapshots, tendon repair involves rapidly evolving spatiotemporal interaction networks for transient events. Novel algorithms and AI-driven approaches may overcome this limitation by enabling predictive modeling of dynamic cellular behaviors.

### 4.1 Clinical translation prospects

Targeting pathological cell subpopulations represents a promising avenue for precision therapy. Tppp3+ progenitors are involved in both tissue repair and ossification (Yea J. H. et al., 2023); selective depletion with anti-CD26 antibody–drug conjugates (ADCs) achieves over a 40% reduction in heterotopic bone volume (Chen S. et al., 2025). In contrast, CRISPR-engineered CAR-T cells directed against PROCR + tenocytes reduce collagen production by 60%, demonstrating therapeutic potential for fibrosis (Chen S. et al., 2025). Looking forward, spatial delivery specificity may be further refined through the use of protease-activated nanocarriers (Ding Z. et al., 2025).

Meanwhile, other strategies center on modulating signaling pathways. Local PI3K-Akt inhibition with AZD5364 directs Tppp3+ progenitors toward tenogenic differentiation, resulting in a 35% increase in tissue tensile strength (Goto A. et al., 2025). Various blocking factors can also prevent mineral deposition in the enthesis. For example, vismogegib-eluting scaffolds inhibit calcification by up to 55%; however, hedgehog pathway inhibition may also compromise normal enthesis development (Feng H. et al., 2020). In addition, both intravesical exosomal fresolimumab administration and systemic TGF-β/SMAD3 inhibition reduce fibrosis progression, though careful dosing is required to avoid immunosuppression (Ng M. T. H. et al., 2024).

Disruption of pathological intercellular communication represents a promising therapeutic strategy. Inhibition of the LGALS9–CD44 axis reduces chondroprogenitor abundance by 65% (Kan C. et al., 2024), and anti-MIF antibodies suppress NF–κB–mediated osteogenesis in translational models (Akbar M. et al., 2021). Chronic inflammation can be targeted with IL-33 decoy receptor–expressing exosomes, which improve collagen alignment by 45% through enhanced IL-33 scavenging.

Overcoming translational barriers requires innovative approaches. Because Tppp3+ markers are expressed by both pathological and reparative cells, refining cell-type specificity with spatial transcriptomics is essential (Fetahu I. S. et al., 2023). The dense extracellular matrix (ECM) of the tendon further restricts delivery, a challenge that may be overcome with MMP13-responsive nanocarriers (Xu Y. et al., 2025). Potential toxicity associated with prolonged pathway inhibition, such as PI3K-Akt, may be reduced by employing pH-sensitive biomaterials (Huang M. et al., 2025). Additionally, species-specific responses underscore the need for validation in patient-derived organoid models (Su W. et al., 2024).

## 5 Conclusion

From a clinical translation perspective, single-cell data-driven therapeutic strategies may focus on two directions: targeting pathological cell subsets and remodeling the repair microenvironment. For aberrant pro-fibrotic or osteogenic cell populations, precision elimination could be achieved via antibody-drug conjugates or CAR-T cell therapies. Additionally, stem cell sorting and expansion technologies based on surface markers may provide high-quality autologous cell sources for transplantation. For microenvironment modulation, engineered exosomes loaded with anti-inflammatory factors, epigenetic regulators, or metabolic reprogramming agents could be delivered via spatiotemporally controlled systems to counteract chronic inflammation or metabolic dysfunction.

In summary, these advancements hold profound theoretical significance for basic science and offer valuable insights for the development of clinical strategies. Single-cell sequencing has unveiled new opportunities in tendon-bone healing research. Future studies should prioritize elucidating dynamic cell-cell interactions to propel transformative progress in this field.

## Abbreviations

AI, artificial intelligence; CITE-seq, Cellular Indexing of Transcriptomes and Epitopes by Sequencing; ECM, extracellular matrix; MSC, mesenchymal stem cell; scRNA-seq, single-cell RNA sequencing; Tppp3, tubulin polymerization-promoting protein family member 3; TSPCs, tendon stem/progenitor cells (TSPCs); Treg, Regulatory T cell.
